# Advocacy, education, and the role of not-for-profit organizations in Lewy body dementias

**DOI:** 10.1186/s13195-014-0059-0

**Published:** 2014-08-28

**Authors:** Angela Taylor, Cecilia Yardley

**Affiliations:** 1 Lewy Body Dementia Association, 912 Killian Hill Road, S.W, Lilburn 30047, GA, USA; 2 Lewy Body Society, Hudson House, 8 Albany Street, Edinburgh EH1 3QB, UK

## Abstract

Lewy body dementias (LBDs) represent a spectrum of dementias that are associated with the presence of Lewy bodies in the brain and that dramatically impact both the person diagnosed and the family caregiver. LBD charities provide education of the public and health-care professionals, emotional support to families, and advocacy to policy-makers on the needs of LBD families and advance research. The US-based Lewy Body Dementia Association and the Lewy Body Society in the UK play an important role in reducing the burden that LBD places on families and society and provide leadership on issues affecting LBD families. Health-care providers are encouraged to refer families upon diagnosis to LBD charities as an additional resource to clinical care.

## Introduction

Lewy body dementias (LBDs) are related brain disorders affecting cognition, motor function, mood, behavior, and autonomic function. Despite being the second most common form of progressive dementia (10% to 15% of all dementia cases) [1], LBD is the most misdiagnosed [2] as onset can present as a psychiatric disorder or Alzheimer’s or Parkinson’s disease. The Lewy Body Dementia Association (LBDA) and the Lewy Body Society (LBS) are the only not-for-profit organizations in the US and Europe, respectively, that focus exclusively on LBD by disseminating information and promoting awareness, providing support for people affected by LBD, advocating on behalf of LBD families, and funding research.

The term ‘LBD’ covers two related clinical diagnoses which have similar underlying pathology and symptoms but which have different patterns of onset. ‘Dementia with Lewy bodies’ (DLB) is diagnosed when a person develops dementia and any other DLB symptoms before, or within a year of, developing extrapyramidal symptoms (bradykinesia, rigidity, or postural instability), with tremor being a less pronounced feature. Many individuals with Parkinson’s disease will go on to develop dementia a year or more after the onset of motor symptoms. This is diagnosed as ‘Parkinson’s disease dementia’ (PDD). The diagnostic criteria for DLB and PDD have many common features. This ‘one-year rule’, though a rather arbitrary boundary, is useful as a guide during diagnosis. The distinction is essential for research purposes, however, and more studies are needed to better understand the expression of Lewy body spectrum symptoms over the course of the disease.

In this article, LBD is used to refer to both clinical diagnoses. DLB or PDD will be used only when referring to a specific clinical diagnosis. There are approximately 1.3 million people in the US and 140,000 in the UK with LBD. At least 75% of people with Parkinson’s disease who survive for more than 10 years will develop dementia [3]. Individuals with LBD can be expected to live 5 to 8 years after diagnosis [4]. Whereas the other articles in this special series of *Alzheimer’s Research & Therapy* will focus on clinical, cognitive, and biomarker characteristics of LBD, this article will focus on the work of two LBD charities (Box 1).

## Education of the public and health-care professionals

Public understanding of LBD lags dramatically behind that of diseases that have been in the public eye for decades longer, such as Parkinson’s and Alzheimer’s diseases. Most individuals with DLB and their families are unaware that the disorder exists, until diagnosis. Parkinson’s disease is still viewed by the general public as largely a motor disorder without cognitive symptoms, although there is growing recognition among health-care providers of its non-motor symptoms.

An LBDA-sponsored survey of 962 LBD caregivers [5] found that eight out of 10 cases of LBD are initially diagnosed as another condition, most often Alzheimer’s, a movement disorder like Parkinson’s, or a psychiatric condition. Lack of an early, accurate diagnosis deprives people of information that explains disturbing symptoms. It can also put them at risk of medication side effects. Up to half of the people with DLB who are treated with antipsychotic medications display severe sensitivity. Medications, like haloperidol, that may be used to control hallucinations in Alzheimer’s disease can have devastating consequences for someone with LBD, hastening disease progression or even causing death.

A definite diagnosis of LBD can only be confirmed post-mortem. Because of the range of cognitive, behavioral, movement, and autonomic symptoms, an incomplete picture of onset symptoms can delay diagnosis. Part of the problem is the use of inadequate assessment tools, such as the Mini-Mental State Examination, which is not sensitive enough to detect cognitive changes in early DLB, such as fluctuating cognition, visuospatial deficits, or attention difficulties. LBD charities provide information to help health-care professionals make earlier, accurate diagnoses, and campaign for the development and adoption of more robust diagnostic tools.

A survey by the LBDA about LBD caregivers’ experience with clinical care indicates that in the US neurologists make nearly two thirds of all LBD diagnoses but that primary care physicians make less than 10% [5]. This indicates a gap in diagnostic capability between specialists and general practitioners. Many primary care doctors are also unfamiliar with the complex diagnostic criteria for DLB. The LBD charities work to raise awareness of LBD among primary care physicians, both directly and through their professional associations, to encourage them to refer anyone who may have LBD to a specialist for diagnosis. Information resources such as the LBDA’s LBD Diagnostic Symptoms Checklist [6] and the LBS’s new information leaflets [7] can increase the knowledge and confidence of frontline staff in working with people with LBD.

Many newly diagnosed people with DLB return to their primary care doctor for follow-up clinical care [5]. Continuing professional medical education is vital to ensure comprehensive treatment. LBD charities urge closer coordination between clinicians who are treating the same patient for different LBD symptoms, because managing the cognitive, motor, and behavioral symptoms of LBD requires a delicate balance in order to relieve one symptom without unintentionally or unduly exacerbating another.

Encouragingly, there are signs that more people are receiving a specific diagnosis of DLB, as ‘In memoriam’ gifts to these condition-specific charities are rising. Both the LBDA and the LBS are driven, however, by the stark reality that there is still a long way to go to educate the public and health-care professionals about the presentation and impact of this disease. Vital efforts are being made to inform the public and the health-care profession about LBD via social media, public service announcements featuring celebrities, paid advertising, and public relations initiatives.

## Outreach and partnership with the Lewy body dementia community

Lifting the burden on LBD families directly or indirectly requires commitment to seek a comprehensive understanding of the challenges created by the disease, not only for the person with the LBD but for the primary caregiver and the immediate family. This understanding is then used to design programs and services that ultimately reduce those challenges (Box 2).

Upon receiving a diagnosis, LBD families face different pathways to education about the condition and referrals to resources for advice, support, and community services. People diagnosed in a specialist dementia or movement disorders clinic are more likely to receive LBD educational information and be referred to organizations like the LBDA and the LBS. UK guidelines for diagnosing dementia make it most likely that diagnosis will take place in secondary care. In the US, however, it is most common for people to be diagnosed by general neurologists and psychiatrists who diagnose and treat patients with a much broader range of neurological or psychiatric disorders. This reduces the likelihood of receiving disease-specific information and referrals. Once diagnosed, most people with LBD return to primary care for symptom management [5]. This underscores the importance of increasing LBD education for primary care physicians.

Although in some practices doctors refer people to LBD charities as a valuable add-on to clinical care, most people find their way to the charities via the internet. Families come seeking both educational information and emotional support from LBD charities, whose staff and volunteers are sometimes the first to answer difficult questions and listen to distraught caregivers. Calls are often received after the initial diagnosis and then at varying times of caregiver stress, including major holidays when extended family come face to face with heart-breaking signs of disease progression or caregiver burnout. Both charities make it clear that the information they supply is not a substitute for advice from a trained professional, but by involving leading experts in the development of information materials, the charities’ resources are both current and quality-ensured.

As an adjunct resource to the clinician, the LBDA and the LBS relieve the strain on the health-care system by helping people understand LBD and directing them to other sources of information. They also give hope to LBD families by providing news about advances in research and increase self-sufficiency by providing advice on caring and access to services.

LBD caregivers report medium to high levels of stress from caring for a person with LBD [8]. Caregiver stress is associated with the presence of psychosis, daytime sleep, and cognitive fluctuations, which are common features in people with DLB and PDD [9]. As LBD progresses, so does the need to understand medication sensitivities, behavioral problems, and long-term care requirements. Most caregivers ask: ‘What does the course of LBD really look like? What can I expect?’ Becoming part of an established LBD community provides access to others with experience and new perspectives about the disease (for example, the impact on the caregiver (and need for self-care) as well as caregiving suggestions for the person with LBD). During late-stage LBD, caregivers often struggle with the emotional realities and practical decisions about end-of-life issues and they value the support of others who have had this experience.

LBD organizations can also encourage people affected by LBD to become active advocates to help raise the profile of the disease. Families often become frustrated when they learn that very few people are familiar with LBD, including many medical professionals. This generates a strong motivation in some individuals to take action through volunteerism to raise awareness and to serve as a resource for other LBD families. Being a knowledgeable advocate about LBD is empowering and helps balance the feeling of powerlessness one has against a degenerative disease.

After the death of a person with LBD, the charities can help families make sense of their experience and draw positive things from it. Recording the name of the deceased on an ‘In memoriam’ webpage or in an online forum can provide solace and community that helps to overcome the isolation and depletion associated with LBD. Some family members actively engage with LBD organizations after a time of grieving. After the devastating impact of LBD on their lives, others need to close the door on LBD, heal, recharge, and move on.

## Advocacy in action

LBD charities advocate for people who are currently underserved. People with LBD may be disenfranchised because of low public awareness, stigma, ageism, insufficient resources, and the application of the medical model instead of person-centered solutions. LBD organizations endeavor to make the issues surrounding and arising from LBD high on the agendas of relevant researchers, clinicians, industries, government agencies, and other organizations concerned with the development and delivery of health-care and social services.

Dementia advocacy within the charity sector has diversified in recent years, as research sheds more light on how different dementias impact individuals and families. The LBDA and the LBS engage with other not-for-profit organizations in advocacy initiatives, acting independently of, in parallel with, or in direct collaboration with related disease-specific groups. LBD charities can participate effectively in broad alliances calling for more funding for key governmental agencies or research funding for dementia and movement disorders. However, LBD charities must also assertively emphasize the distinctive needs of their beneficiaries (for example, the development of safer medications to treat behavioral problems in LBD).

LBD charities are increasing their presence among government bodies, bringing to their attention the challenges and needs arising from and surrounding LBD. Those affected by LBD are now being represented at national and international research strategy meetings for dementia and Parkinson’s disease. Other advocacy activities include testifying to drug regulatory agencies about the importance of approving drugs specifically for LBD symptoms, speaking out for early disability benefits for people with LBD, and supporting the development of new psychiatric codes for LBD in order to improve treatment.

Although LBD charities have been formed in other countries, including Argentina, Australia, and Japan, people outside the US and the UK frequently seek information and support from the LBDA and the LBS because of the lack of an equivalent organization in their own countries. More LBD charities are needed around the globe to advocate on behalf of LBD families and to increase synergistic opportunities for research advances. The LBDA and the LBS have served as useful advisors and resources to emerging LBD charities.

## Advancing research

Supporting research is a primary objective for both LBD charities, as the greatest need of those affected by LBD is for clinical advances leading to better treatments and ultimately a cure.

LBD charities place vital donated funds in the hands of researchers in the form of grant awards. Other research programs include caregiver research, fellowships, and convening scientific meetings. Collaborations with government agencies, industry, and related disease charities are imperative to minimize research silos as well as build synergy and minimize duplication of effort.

## Conclusions

The LBDA and the LBS deliver a wide range of services to lift the burden of LBD on families and society. By providing support and promoting awareness, LBD charities reduce the personal distress experienced by LBD families and may reduce excessive use of the health system. The LBS and the LBDA offer resources that enable health-care professionals to make earlier, accurate diagnoses, and promote better understanding of the condition among the public, clinical and care professions, and the many agencies that serve the LBD community.

Individuals personally touched by LBD and leaders in relevant fields drive the work of both organizations, and LBD charities ensure that strategies, programs of research, and services are quality-ensured by LBD experts. The needs of LBD families evolve as the condition advances and the charities offer continuity of contact as individuals move through the health and social care system. They also sustain a community of people who understand the struggles of others affected by LBD. By offering a range of opportunities to find meaning and purpose, the charities help people overcome their sense of powerlessness in the face of a degenerative disease.

Families seeking information and emotional support currently find their way to the LBDA and the LBS via the internet, rather than referral from a health or social care professional. Providers are encouraged to refer families upon diagnosis to LBD charities as an additional resource to clinical care.

## Note

This article is part of a series on *Lewy Body Dementia*, edited by Ian McKeith and James Galvin. Other articles in this series can be found at http://alzres.com/series/LewyBodyDementia

## **Box 1. Lewy body dementia charities, driven by experience**

The Lewy Body Dementia Association (LBDA) was formed in 2003 by a group of family caregivers in the US and is raising awareness of Lewy body dementia (LBD) in the public and health-care community, providing outreach and education to LBD families, and advancing research. The LBDA’s Scientific Advisory Council members are leaders in LBD research and clinical management who provide strategic input to the LBDA’s programs and services. The LBDA engages its LBD Community Discussion Panel of caregivers and individuals with LBD to ensure that its programs and services are designed to meet the evolving needs of families affected by LBD.

The Lewy Body Society was founded in the UK in 2006 and its aims are to support research into dementia with Lewy bodies and to raise awareness and educate the general public, the medical profession, and relevant decision-makers about all aspects of the disease. It is the only organization in Europe dedicated to dementia with Lewy bodies and has strong links with Parkinson’s UK, a charity advocating on behalf of Parkinson’s disease. Caregiver support is through provision of information via the website, written materials, and response to enquiries. The Lewy Body Society Specialist Advisory Committee consists of 11 medical, scientific, and legal experts with a special interest in LBD.

## **Box 2. Lewy Body Dementia Association services**

The programs and services of the Lewy Body Dementia Association (LBDA) reflect its corporate tagline, ‘Increasing Knowledge, Sharing Experience, Building Hope’ and provide outreach to families, increase awareness in the public and health-care profession, and foster research. Family services include a national network of caregiver support groups, virtual Lewy body dementia (LBD) communities, and a Caregiver Link [10] which connects caregivers in the US to volunteers with experience in caring. Educational activities include free publications and webinars on LBD and related caring. The LBDA publishes a monthly e-newsletter reporting on LBD research and resources for families. The website [11] features news, educational resources, stories from LBD families, and tools, such as a Speakers’ Kit, for raising awareness. The LBDA fosters research by organizing scientific conferences, awarding grants for pilot studies, and evaluating the impact of LBD on the family caregiver.

Lewy Body Society services

The initiatives of the Lewy Body Society (LBS) embody its slogan, ‘The more people who know, the fewer people who suffer’ and its programs center on raising awareness and promoting and funding research. Information is provided through written materials and a comprehensive website [12]. Queries are answered by post, email, and telephone, and where appropriate, referrals are made to other organizations or agencies. The LBS helpline is answered by nurses with expertise in dementia through its partnership with Parkinson’s UK. The LBS sponsors research projects in the genetics of LBD and the development of new drugs for dementia with Lewy bodies [13] and is currently seeking to extend its research portfolio.

## Abbreviations

DLB: Dementia with Lewy bodies

LBD: Lewy body dementia

LBDA: Lewy body dementia association

LBS: Lewy body society

PDD: Parkinson’s disease dementia

## Competing interests

AT and CY are employed by the LBDA and the LBS, respectively.
